# Identification of a Novel Mutation in *BRD4* that Causes Autosomal Dominant Syndromic Congenital Cataracts Associated with Other Neuro-Skeletal Anomalies

**DOI:** 10.1371/journal.pone.0169226

**Published:** 2017-01-11

**Authors:** Hyun-Seok Jin, Jeonhyun Kim, Woori Kwak, Hyeonsoo Jeong, Gyu-Bin Lim, Cha Gon Lee

**Affiliations:** 1 Department of Biomedical Laboratory Science, College of Life and Health Sciences, Hoseo University, Asan, Chungnam, Republic of Korea; 2 Department of Medical Genetics, Ajou University School of Medicine, Suwon, Republic of Korea; 3 C&K Genomics, Seoul, Republic of Korea; 4 Department of Animal Sciences, University of Illinois, Urbana, Illinois, United States of America; 5 Department of Pediatrics, Nowon Eulji Medical Center, Eulji University, Seoul, Republic of Korea; Tsinghua University School of Life Sciences, CHINA

## Abstract

Congenital cataracts can occur as a non-syndromic isolated ocular disease or as a part of genetic syndromes accompanied by a multi-systemic disease. Approximately 50% of all congenital cataract cases have a heterogeneous genetic basis. Here, we describe three generations of a family with an autosomal dominant inheritance pattern and common complex phenotypes, including bilateral congenital cataracts, short stature, macrocephaly, and minor skeletal anomalies. We did not find any chromosomal aberrations or gene copy number abnormalities using conventional genetic tests; accordingly, we conducted whole-exome sequencing (WES) to identify disease-causing genetic alterations in this family. Based on family WES data, we identified a novel *BRD4* missense mutation as a candidate causal variant and performed cell-based experiments by ablation of endogenous *BRD4* expression in human lens epithelial cells. The protein expression levels of connexin 43, p62, LC3BII, and p53 differed significantly between control cells and cells in which endogenous *BRD4* expression was inhibited. We inferred that a *BRD4* missense mutation was the likely disease-causing mutation in this family. Our findings may improve the molecular diagnosis of congenital cataracts and support the use of WES to clarify the genetic basis of complex diseases.

## Introduction

A cataract is an opacity of the eye lens that causes partial or total blindness. Congenital cataracts presents at birth or during early childhood. Cataracts are a common and often curable cause of blindness in children [1]. The prevalence of congenital cataracts in developed countries is 1–3 in 10,000 [2–4]. The pathogenic mechanisms of cataract formation in infancy and childhood are not yet fully understood [5]. However, approximately 50% of all congenital cataract cases have a heterogeneous genetic basis [6,7]. Congenital cataracts can be isolated, accompanied by other ocular disorders, or occur as a part of genetic syndromes in multi-systemic diseases, such as Nance–Horan syndrome (MIM 302350) and Marinesco–Sjogren syndrome (MIM 248800) [8].

Hereditary cataracts are typically autosomal dominant with almost complete penetrance, but with variable expression. Autosomal recessive and X-linked forms are less frequent [7]. Only a few years ago, more than 26 disease loci or genes were described in the literature [6,7,9,10]. Currently, mutations in more than 50 genes causing congenital cataracts have been reported in the Human Gene Mutation Database and by Behnam et al. [11]. This rapid increase in the number of causal genes for congenital cataracts has been possible owing to advances in DNA sequencing technology, especially whole exome sequencing (WES), which enable clinical researchers to discover rare disease-causing genes.

Here, we describe three generations of a family with autosomal dominant syndromic congenital cataracts associated with systemic neuro-skeletal anomalies. First, we performed karyotyping and array comparative genomic hybridization analyses. We did not detect any abnormalities using these approaches. Accordingly, we examined the family by WES. Among several candidate mutations, we focused on a *BRD4* mutation (c.910C>T, p.His304Tyr) and performed cell-based experiments by ablation of endogenous *BRD4* expression in human lens epithelial cells. Finally, we inferred that *BRD4* is a novel causative gene of congenital cataracts.

## Materials and Methods

### Ethics statement

The Institutional Review Board of Eulji General Hospital in Seoul, Korea (IRB #2014-06-007-001) approved the use of human clinical materials and blood in this study. Written informed consent for genetic testing was obtained from all subjects before participation.

The animal research procedures were approved by the Animal Care and Use Committee of the Ajou University School of Medicine (IACUC No. 2014–0066), and all experiments were conducted in accordance with the institutional guidelines established by the Committee. All efforts were made to minimize animal suffering and to reduce the number of mice used.

### Subjects

**Fig 1:**
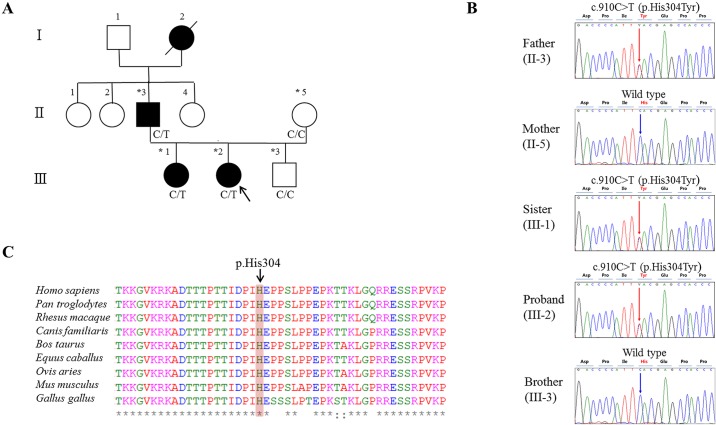
Pedigree and Sanger sequencing of the family with congenital cataracts and amino acid sequence conservation. (A) Pedigree of the family. The three generations of the family comprised four affected members (I-2, II-2, III-1, and III-2) and six unaffected members. The black arrow indicates the proband (III-2). Darkened symbols represent affected members. Stars indicate sampled subjects. Genotypes at the *BRD4* mutation site are indicated below each examined family member. (B) Sequencing chromatograms, vertical arrows indicate the mutation site. Sanger sequencing confirmed the *BRD4* heterozygous mutation (c.910C>T, p.His304Tyr) in all patients (II-3, III-2, and III-1) and the wild-type genotype in the unaffected family members (II-5 and III-3). (C) Conservation analysis of the amino acid sequence; the mutation site was highly conserved across vertebrate species.

Three generations of a family with congenital cataracts and neuro-skeletal anomalies were identified at the Department of Pediatrics, Eulji Medical Center (Seoul, Republic of Korea). The pedigree is illustrated in Fig 1. Three generations of this family comprised four affected members (I-2, II-3, III-1, and III-2) and six unaffected members. The proband was the younger sister (III-2). Physical examinations and laboratory/imaging tests of all affected members were performed. DNA was obtained from five family members (patients: II-3, III-1, and III-2; controls: II-5 and III-3). To identify the causative genetic alterations in the index patient (III-2), G-band karyotyping was performed to detect chromosomal aberrations and an array comparative genomic hybridization analysis was performed to detect copy number variation using the Roche NimbleGen CGX-3 135K Whole-Genome Array (Roche NimbleGen, Inc., Madison, WI, USA). However, abnormalities were not detected using these approaches.

### Whole exome sequencing

WES was performed for four family members (patients: II-3 and III-2; control: II-5 and III-3) out of five members who agreed to provide a blood sample (II-3, III-1, III-2, II-5, and III-3) to identify the disease-causing mutation(s). Three micrograms of fragmented DNA were prepared to construct libraries using the TruSeq Exome Enrichment Kit (Illumina, Inc., San Diego, CA, USA) according to the manufacturer’s protocol. Briefly, the qualified genomic DNA sample was randomly fragmented using a Covaris instrument, followed by adapter ligation, purification, hybridization, and PCR. Captured libraries were evaluated using the Agilent 2100 Bioanalyzer (Agilent Technologies, Waldbronn, Germany) to estimate quality and were loaded on the Illumina HiSeq2000 according to the manufacturer’s recommendations. Raw image files were processed using HCS1.4.8 for base-calling with default parameters. For each individual, 101-bp paired-end reads were obtained.

### Data analysis and variant calling

The quality of the WES reads from all samples was checked using FastQC [12] and a raw data quality control process was conducted using Trimmomatic [13] with the following parameters: ILLUMINACIP: TruSeq3-PE-2.fa:2:30:10, MINLEN: 75. Reads retained after these basic quality control processes were used for subsequent analyses. Paired-end reads of each sample were mapped to the human reference genome (hg19) using Bowtie2 [14]. Using the “REMOVE_DUPLICATES = true” option in the “MarkDuplicates” command-line tool of Picard (http://broadinstitute.github.io/picard, v.1.109), potential PCR duplicates were removed. SAMtools [15] was used to create index files for reference and BAM files. Genome Analysis Toolkit (GATK) [16] was used for downstream processing and variant calling. Local realignment was conducted using GATK to correct misalignments due to the presence of indels. The “UnifiedGenotyper” of GATK was used to call candidate single nucleotide variants (SNVs) and indels. SNPs and indels were annotated according to genomic region (Intergenic, 5′ UTR, Intron, CDS, Splicing, or 3′ UTR). Candidate variants were selected based on frequency, inheritance model, pathogenicity, and clinical phenotype. Non-causative variants were filtered based on population frequency (homozygous in fewer than 0.5% of individuals) using various databases (e.g., dbSNP build 138, 1000 Genomes, and ExAC). Rare nonsynonymous variants with suitable autosomal-dominant inheritance patterns based on our family were selected. To predict whether an amino acid substitution will affect protein function, variants were annotated using disease mutation databases [e.g., Online Mendelian Inheritance in Man (OMIM) and Human Gene Mutation Database (HGMD)] and protein function prediction tools [PolyPhen2 (Polymorphism Phenotyping v2), Provean (Protein Variation Effect Analyzer), and MutationTaster].

### Variant confirmation

Sanger sequencing was performed to determine whether the variants detected by WES co-segregated with the disease phenotype in this family. Five individuals (II-3, III-2, III-1, II-5, and III-3) were examined. Genomic DNA was isolated from peripheral whole blood leukocytes and genes were amplified by PCR. The PCR product was sequenced using an ABI 3500xL DNA Analyzer (Applied Biosystems, Foster City, CA, USA). DNA sequencing was performed at the DNA Sequencing Center (Macrogen, Seoul, Korea).

### Cell culture

Human lens epithelial cells (HLE-B3; American Type Culture Collection, Manassas, VA, USA) were grown in Dulbecco’s Modified Eagle Medium (Thermo Fisher Scientific Inc., Waltham, MA, USA) supplemented with 10% fetal bovine serum (Sigma-Aldrich; St. Louis, MO, USA), 100 U/ml penicillin (Duchefa; RV Haarlem, the Netherlands), and 100 μg/ml streptomycin (Duchefa). HLE-B3 cells were infected with sh*BRD4* (Sigma Aldrich) viral particles for 24 h. All cultured cells were incubated at 37°C in a humidified atmosphere containing 5% CO_2_.

### Reverse-transcription polymerase chain reaction (RT-PCR)

Total RNAs from human lens epithelial B3 cells were isolated using TRIzol reagent (Invitrogen, Carlsbad, CA, USA) and treated with RNase-free DNase I (Invitrogen). Total RNA (1 μg) was subsequently reverse-transcribed using the RevertAid^™^ Minus First Strand cDNA Synthesis Kit (Fermentas Inc., Hanover, MD, USA) with both the oligo (dT) 15–18 primer and a random hexamer primer. PCR amplification (25 cycles) was performed in a total volume of 20 μl containing 100 ng of cDNA using the i-MAX II DNA Polymerase Kit (iNtRON Biotechnology, Gyeonggi, Korea) according to the manufacturer's recommendations. The following specific primers were used: 5′-GGT GCA CAT CAT CCA GTC ACG-3′ and 5′-GGA GCC GGC AAT CAC ATC AAC-3′ for *BRD4* (GenBank: NM_058243.2), and 5′-TGT TGC CAT CAA TGA CCC CTT-3′ and 5′-CTC CAC GAC GTA CTC AGC G-3′ for *GADPH* (GenBank: NM_002046.5).

### Western blotting

Two male C57BL/6J mice (8 weeks old) were obtained from Central Lab. Animal Inc. (Seoul, Korea). Mice were raised under specific pathogen-free conditions, with a 12-h light/12-h dark cycle, and provided free access to a commercial pellet diet. After mice were euthanized by CO_2_ exposure, tissue samples were obtained. The homogenized mouse tissues and HLE-B3 cells were lysed in RIPA buffer (150 mM NaCl, 1% Nonidet P-40, 0.5% sodium deoxycholate, 0.1% SDS, and 50 mM Tris buffer, pH 8.0). The lysates were centrifuged at 16,000 × *g* for 20 min at 4°C to remove cellular debris. The protein concentration of the lysates was determined using the Dc Protein Assay (Bio-Rad Laboratories, Hercules, CA, USA). The extracts were separated by 8% SDS-PAGE and transferred to a PVDF membrane for blotting (Millipore Corporation, Billerica, MA, USA). The membrane was blocked with 5% (w/v) nonfat dried milk, incubated with primary and secondary antibodies, and then visualized using the WEST-ZOL plus ECL Western Blotting Detection System (GenDEPOT, Harris, TX, USA). The following antibodies were used: anti-BRD4 (Abcam, Cambridge, UK), anti-CX43 (Millipore), anti-LC3B and p62 (BD Transduction Laboratories, Lexington, KY, USA), p53 and anti-β-ACTIN (Santa Cruz Biotechnology; Santa Cruz, CA, USA), HRP-conjugated goat anti-mouse IgG, HRP-conjugated goat anti-rabbit IgG, and HRP-conjugated mouse anti-goat IgG antibodies (GenDEPOT).

### Water-soluble tetrazolium salt assay

HLE-B3 cells (3 × 10^3^ cells/well) were placed in 96-well plates overnight. An EZ-Cytox Cell Viability Assay Kit (Daeil, Seoul, Korea) was used for the cell viability assay. Water-soluble tetrazolium salt (WST; 10 μl/100 μl of medium) was added to each well and incubated for additional 1 h at 37°C. The absorbance was then measured at 450 nm and 655 nm using an iMark^™^ microplate reader (Bio-Rad Laboratories).

### Immunofluorescence

To determine the cellular location of endogenous BRD4, HLE-B3 cells were fixed with 4% paraformaldehyde (pH 7.4) for 30 min at room temperature. After washing with cold phosphate-buffered saline (PBS), the fixed cells were incubated with a permeabilization agent including 0.1% Triton X-100 in PBS for 10 min and subsequently incubated with 1% BSA in PBS with Tween 20 (PBST) for 30 min at room temperature. HLE-B3 cells were incubated in a solution containing an anti-BRD4 antibody diluted in 1% BSA-PBST (1:200) overnight at 4°C. Following three rinses with cold PBST, the cells were incubated with an anti-rabbit IgG (FITC) antibody (GenDEPOT) diluted in 1% BSA-PBST (1:500) for 1 h at room temperature, and stained with DAPI to visualize nuclei. BRD4-knockdown HLE-B3 cells were transfected with DsRed-mito or DsRed-F-actin for 24 h to evaluate changes in the distribution of mitochondria and F-actin. DAPI (nuclei), FITC (BRD4), and DsRed (mitochondria or F-actin) were visualized using an excitation wavelength of 350 nm, 488 nm, or 590 nm and an emission wavelength of 540 nm, 519 nm, or 617 nm with a fluorescence microscope (Carl Zeiss, Oberkochen, Germany).

## Results

### Clinical description

**Fig 2:**
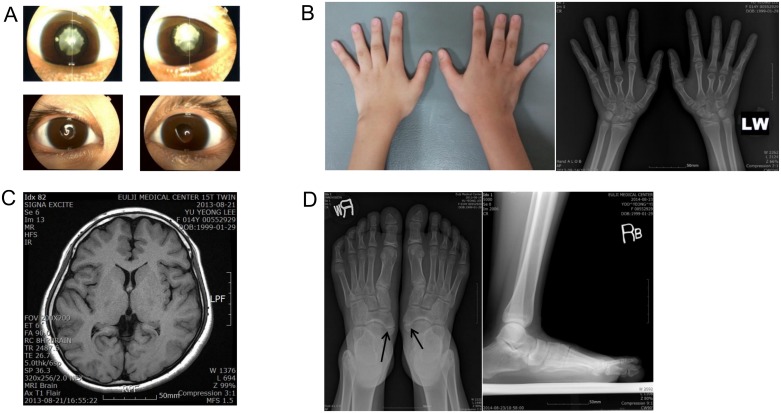
Clinical findings of the proband and preoperative eyes of the father. (A) In the upper panel, both of the father’s eyes showed white congenital cataracts before preoperative slit-lamp images were obtained, and the lower panel shows slit-lamp images for both of the proband’s eyes, showing postoperative bilateral phacoemulsification with posterior chamber lens implantations. (B) Brachydactyly on both hands was observed and hand X-rays revealed short 3rd and 4th left metacarpals and a short right 3rd metacarpal. (C) Macrocephaly without definite brain anomalies on 1.5-tesla magnetic resonance imaging. (D) Foot radiographs showing flat feet and both accessory navicular bones.

The proband was a 15-year-old girl (III-2) who visited for recurrent headaches in December 2012. She received a clinical genetic evaluation on January 2013. She was born at term with a birth weight of 3,500 g as the second child of non-consanguineous Korean parents. She was diagnosed with congenital cataracts within a few days after birth. Her eyes had cortical opacity in the lenses. She also had congenital horizontal nystagmus. She underwent bilateral phacoemulsification for cataracts and posterior chamber lens implantation at 4 years of age in an ophthalmology clinic in May (left eye) and June (right eye) of 2003. After cataract surgery, her current best-corrected visual acuity was 0.2 in both eyes. She did not show any other eye disorders, such as strabismus, other than mild congenital horizontal nystagmus. She exhibited macrocephaly (55.5 cm, 3.15 SDS). She was short in stature; her height was 149.7 cm (-1.99 SDS). Extremities showed shortened 3^rd^ and 4^th^ fingers (Fig 2B) and flat feet (Fig 2D). A radiological skeletal survey showed brachydactyly of the 3^rd^ and 4^th^ metacarpals in the left finger and 3rd metacarpal in the right finger (Fig 2B). She had accessory navicular bones in both feet (Fig 2D). All laboratory tests, chest radiograph, abdomen radiograph, electrocardiography, brain magnetic resonance imaging, and electroencephalogram were normal (Table 1).

**Table 1:** Summary of clinical data for the affected patients in the study family.

Trait	III-2 (Proband)	II-3 (Father)	III-1 (Elder sister)
**Age/sex**	15 years/female	47 years/male	16 years/female
**Birth history**	Term AGA (3500 g)	Unknown	Preterm (36 wk) AGA (2880 g)
**Head size**	Macrocephaly (HC 55.5 cm, 3.15 SDS)	Macrocephaly (HC 58.5 cm, 3.92 SDS)	Macrocephaly (HC 54.5 cm, 2.58 SDS)
**Eye**	CC, congenital horizontal nystagmus	CC, congenital horizontal nystagmus	CC, congenital horizontal nystagmus
**Short status**	Ht 149.7 cm (-1.99 SDS)	Ht 161.5 cm (-2.14 SDS)	Ht 152.5 cm (-1.53 SDS)
**Skeletal anomalies**			
**Hand**	Brachydactyly 3^rd^ and 4^th^ metacarpals	Brachydactyly, Exostosis at distal phalangeal tip of 4th finger, Lt.	Brachydactyly, short of both 5th middle phalanges, clinodactyly
** Foot**	Accessory navicular bones, flat feet	Both hallux valgus, flat feet, Os intermetatarseum, and Enthesophytes at calcaneal attachment site of Achilles tendons	Accessory navicular bones, flat feet
**Brain MRI/EEG**	Normal/normal	Not performed	Not performed
**ECG**	Normal sinus rhythm	Normal sinus rhythm	Normal sinus rhythm
**Laboratory test**	Normal (included thyroid panel, pituitary function, lipid panel and urinalysis)	Normal	Normal

AGA: appropriate for gestational age; HC: head circumference; Bwt: body weight; Ht: height; HC: head circumference; BMI: body mass index; SDS: standard deviation score; CC: congenital cataract; MRI: magnetic resonance imaging; EEG: sleep electroencephalogram; ECG: electrocardiography.

The patient's father (II-3) showed white cataracts in both lenses (Fig 2A). He also had congenital horizontal nystagmus. He underwent bilateral phacoemulsification with posterior chamber lens implantation at age 46 in our ophthalmology clinic in 2013. He also showed macrocephaly (HC 3.92 SDS) and short stature (Ht -2.14 SDS) with minor skeletal anomalies (Table 1).

The patient's older sister (III-1) was diagnosed with congenital cataracts at age four. Her right eye showed cortical opacity and her left eye revealed total opacity in the lens. She also had horizontal nystagmus. She underwent bilateral phacoemulsification with posterior chamber lens implantation at age six in the ophthalmology clinic in May (right eye) and June (left eye) of 2003. She suffered complications after cataract surgery, including posterior capsule opacification, esotropia, intraocular lens dislocation, and recurrent retinal detachment. She underwent further surgery for the complications. She had macrocephaly (HC 2.58 SDS) and a short stature (Ht -1.53 SDS) with minor skeletal anomalies. Detailed information for the other affected patients is summarized in Table 1.

### Whole-exome sequencing analysis

The genomic DNA of four family members (patients: II-3 and III-2, normal: II-5 and III-3) was subjected to WES. An average of 67 million reads passed the quality control thresholds and 99.5% were aligned to the human reference sequence. With respect to coverage depth, 96.6% of the bases were read at least five times and 92.6% of the bases were read at least 10 times. The sequence data and quality control results are summarized in S1 Table.

Based on the family history, we inferred that the proband likely had an autosomal dominant genetic trait. We anticipated finding a heterozygous disease-causing mutation. The causal variant was expected to be heterozygous in all affected members (II-3 and III-2) and must not occur in a reference homozygous state in any of the unaffected individuals (II-5 and III-3).

After data filtering, we identified 225 variants consistent with autosomal dominant inheritance (SNV: 155, deletion: 31, insertion: 39). Among these 225 variants, 67 were located in CDS regions and 42 were nonsynonymous SNPs. There were no pathological mutations in known genes associated with non-syndromic congenital cataracts, like *CRYB*, *CRYG*, *Cx43*, *Cx46*, *Cx50*, *MIP*, *PITX3*, *MAF*, *HSF4*, and so on. Hence, we further investigated candidate genes with respect to their function and cataractogenesis-related cell metabolism, including data from literature searches. Finally, we selected five candidate genes (*BRD4*, *HTT*, *ANPEP*, *WFS1*, and *ZNF274*). Among these five filtered heterozygous mutations, the *Brd4* c.910C>T (p.His304Tyr) variant was strongly supported as a disease-causing mutation based on its absence from controls in dbSNP build 138, 1000 Genomes, ExAC, and Korean Reference Genome Database (KRGdb), multiple *in silico* protein function predictions, and a literature search (Table 2). The identity and heterozygosity of this mutation were confirmed by PCR amplification and direct Sanger sequencing of all family members (II-3, III-2, III-1, II-5, and III-3), as shown in Fig 1B. No loss-of-function mutations have been reported in normal human *BRD4*. This site was highly conserved among vertebrate species (Fig 1C). A previous study indicated that Brd4-deficient mice exhibit syndromic abnormalities, including cataracts, similar to the symptoms observed in patients in this study [17].

**Table 2:** Summary of five candidate variants with a consistent inheritance pattern detected by whole exome sequencing analyses.

Gene	Accession number	Nucleotide change	AA change	Polyphen2	Provean	MutationTaster	AF of KRGdb ^a^
*BRD4*	NM_058243.2	c.910C>T	p.His304Tyr	Possibly damaging	Deleterious	Disease-causing	0
*HTT*	NM_002111.7	c.1652G>A	p.Gly551Glu	Probably damaging	Neutral	Disease-causing	0.045
*ANPEP*	NM_001150.2	c.694G>A	p.Glu232Lys	Benign	Neutral	Polymorphism	0
*WFS1*	NM_001145853.1	c.41A>G	p.Gln14Arg	Benign	Neutral	Polymorphism	0.014
*ZNF274*	NM_133502.2	c.317T>C	p.Met106Thr	Benign	Neutral	Polymorphism	0

^a^ Korean Reference Genome (KRG) project involved whole genome sequencing for 622 Korean individuals

AA: amino acid; AF: allele frequency; KRGdb: Korean Reference Genome database; PolyPhen 2: Polymorphism Phenotyping v2; Provean: Protein Variation Effect Analyzer.

Unfortunately, additional families or individuals with congenital cataracts harboring the same mutation or other mutations in *BRD4* were not found.

### *BRD4* expression profile in mouse tissues

**Fig 3:**
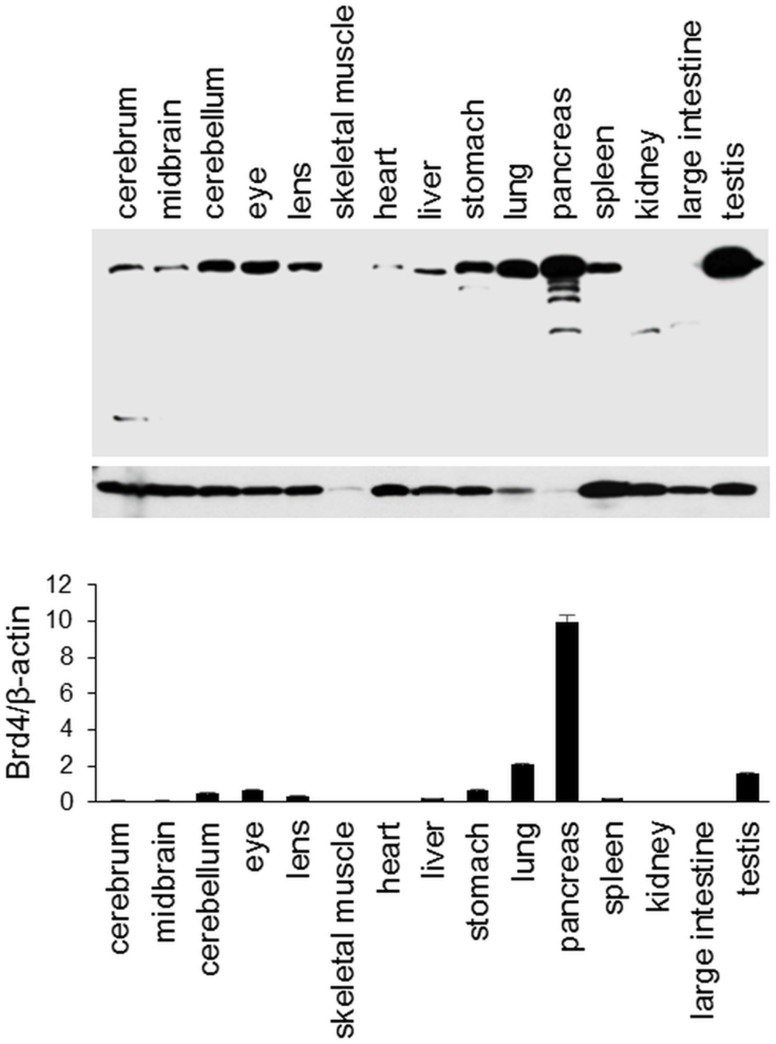
BRD4 protein expression profile in mouse tissues obtained from two male mice. Protein levels of BRD4 and β-actin were analyzed by western blotting and protein quantities were determined using ImageJ (National Institutes of Health, Bethesda, MD, USA) by densitometry. Relative ratios of the Brd4/β-actin proteins are shown.

The *BRD4* gene is expressed at high or medium levels in various tissue types, including the brain, muscle, and skeletal tissues, according to GTEx Analysis Release V4. However, no information is available regarding eye tissue-specific expression of *BRD4*. We examined the expression patterns of *BRD4* in mouse tissues, including eye tissues. The gene product showed high variation in expression among tissue types. Based on a western blot analysis, *BRD4* expression was also found in the eye, including the lens, as shown in Fig 3.

### Cell function analyses of BRD4

**Fig 4:**
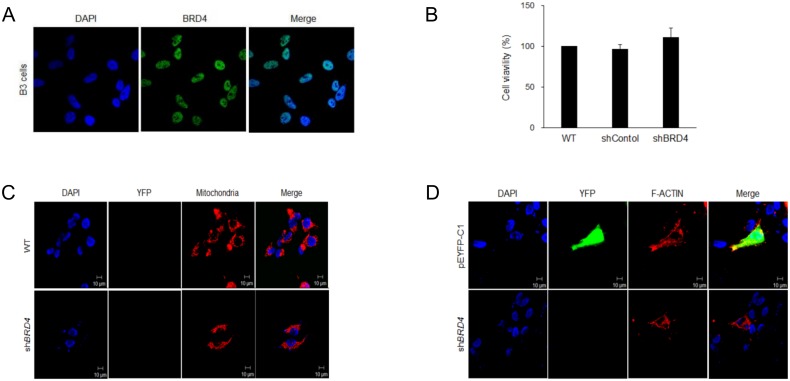
Cell function analyses of *BRD4*. **(**A) The cellular location of BRD4 in HLE-B3 cells, (B) the effect of the ablation of endogenous *BRD4* expression on cell viability, (C) changes in mitochondrial morphology induced by shBRD4, (D) and the influence of shBRD4 on F-actin.

We examined the cellular location of endogenous BRD4 in HLE-B3, which are human lens epithelial cells. The immunocytochemistry results indicated that BRD4 is mainly located in the nuclear region (Fig 4A). We investigated the effects of the gene on cell viability by the ablation of endogenous *BRD4* expression using shBRD4. However, the inhibition of *BRD4* expression did not influence cell viability (Fig 4B).

A previous study indicated that a mouse model with congenital nuclear cataracts exhibits a loss of cytoskeleton integrity and mitochondrial accumulation [18]; accordingly, changes in F-actin and mitochondria were also examined. However, the inhibition of BRD4 expression did not alter either property, as shown in Fig 4C and 4D.

### Effects of the ablation of BRD4 on cataractogenesis-related proteins in lens cells

**Fig 5:**
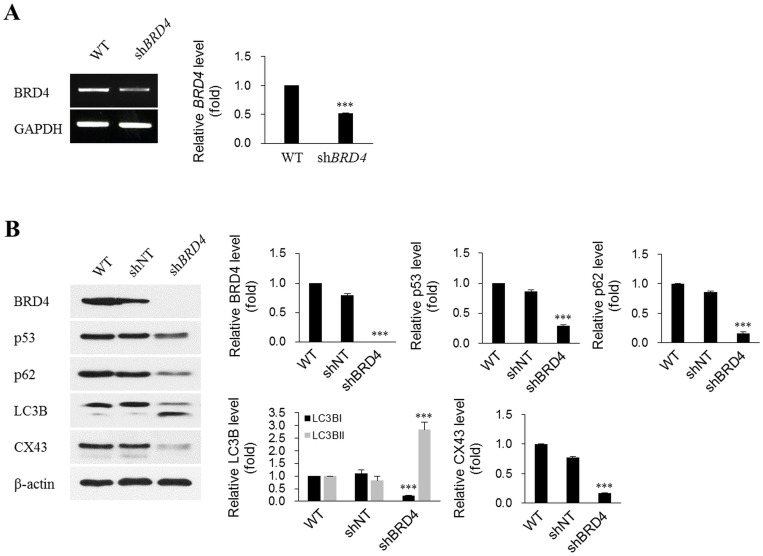
Ablation of endogenous BRD4 expression in HLE-B3 cells using shRNA. HLE-B3 cells were not transfected (wild-type), mock-transfected (shNT), or transfected with shRNA targeting *BRD4* (shBRD4) for 48 h, and were then subjected to RT-PCR (A) and western blotting (B) to detect BRD4 mRNA and protein levels. β-Actin and GAPDH served as loading controls. ShBRD4 significantly ablated endogenous BRD4 at both the mRNA and protein levels. Decreases in p53, p62, LC3BI, and CX43 protein levels, and an increase in LC3BII were detected after BRD4 knockdown. Protein levels were analyzed by western blotting. Western blots from three independent experiments were quantified and normalized against β-actin. The quantities were determined using ImageJ (National Institutes of Health) and all data were normalized to β-actin. ***p < 0.001 vs. wild type.

Autophagy is a major cellular homeostasis mechanism and is related to congenital nuclear cataract [18]. Mutations in gap junction proteins, which mediate interactions with adjacent cells, are also causes of congenital cataracts [9]. Moreover, BRD4 binds to p53 as a functional partner [19]. Therefore, we examined the effect of BRD4 expression on proteins related to autophagy, gap junctions, and p53. The protein quantities of connexin 43 (CX43; gap junction alpha-1 protein), p62 (sequestosome 1, SQSTM1), and p53 were substantially decreased by shBRD4 transfection. However, the quantity of LC3BII (microtubule-associated protein 1 light chain 3 beta, MAP1LC3B) was significantly increased, as shown in Fig 5B. The expression changes observed for p62 and LC3BII indicate an association between the ablation of BRD4 expression and autophagy.

## Discussion

Many genes are involved in syndromic and non-syndromic congenital cataracts, with different inheritance patterns [10,20,21]. Mutations in distinct genes that encode the main cytoplasmic proteins of the human lens are associated with cataracts of various morphologies. These genes include those encoding crystallins (*CRYAA*, *CRYAB*, *CRYBA1*, *CRYBB1*, *CRYBB2*, *CRYBB3*, *CRYGC*, and *CRYGD*), membrane proteins (the gap junction protein-alpha 3 gene *GJA3*; the gap junction protein-alpha 8 gene *GJA8*; and the major intrinsic protein of lens fiber gene *MIP*), a cytoskeletal protein (the beaded filament structural protein 2 gene *BFSP2*), growth and transcription factors (the v-maf avian musculoaponeurotic fibrosarcoma oncogene homolog gene *MAF*), and other proteins (fibrosarcoma oncogene homolog gene *MAF*; ferritin light chain gene *FTL*; galactokinase 1 gene *GALK1*; FYVE and coiled–coil domain-containing 1 gene *FYCO1*; and Nance–Horan gene *NHS*) [6,7,9,10]. A representative example of syndromic congenital cataracts is Nance–Horan syndrome, a rare X-linked cataract disease associated with dental anomalies and facial dysmorphia [22].

In this study, we identified a novel *BRD4* mutation in a three-generation family that had autosomal dominant congenital cataracts, macrocephaly, short stature, and minor skeletal anomalies, including brachydactyly and flat feet. The present study included one familial case with three affected individuals; additional families or individuals with congenital cataracts harboring mutations in *BRD4* were not found. The causal link between the *BRD4* mutation and congenital cataracts was supported by *in vitro* functional analyses using human lens epithelial cells, including analyses of the effects on autophagy, gap junctions, and p53 protein expression.

Bromodomain-containing protein 4 is encoded by the *BRD4* gene in humans. *BRD4* belongs to the adaptor protein family comprised of *BRD2*, *BRD3*, *BRD4*, and *BRDT*, which perform diverse roles in transcriptional regulation by RNA polymerase II (Pol II) [23]. All four BET proteins have two conserved N-terminal bromodomains (BD1 and BD2), which are chromatin interaction modules that recognize acetylated lysine residues on histone tails and other nuclear proteins [24]. Bromodomain-mediated interactions with acetylated chromatin result in the localization of BET proteins to discrete positions along the chromosome, where they recruit other regulatory complexes to influence gene expression [23,25]. A well-studied member of the BET family is *BRD4*, whose role in general transcriptional regulation was initially suggested by the constituents of its associated protein complex [25,26].

Houzelstein et al. [17] performed functional analyses of the mouse *BRD4* gene. It is located on mouse chromosome 17 and has conserved synteny with human chromosome 19p13.1. They demonstrated that *BRD4* mice exhibit early embryonic lethality. In addition, they revealed that a single functional *BRD4* allele is not able to sustain normal development. The most remarkable phenotype in *BRD4* heterozygous mice is their pre- and post-natal growth defects. They anticipated that this phenotype is associated with haploinsufficiency, and heterozygous mutations in the *Brd* human homologues could also be associated with pre- and post-natal growth defects. Therefore, *Brd* loci may be candidates for human disorders characterized by a short stature. These mice also exhibit a variety of additional anatomical abnormalities, e.g., head malformations, an absence of subcutaneous fat, cataracts, and abnormal liver cells. This phenotype of the *BRD4* heterozygous mouse mutant is highly similar to that of our patients, including the short stature, cataracts, and brain malformation.

We focused on the effect of the inhibition of endogenous *BRD4* expression owing to the lack of studies supporting a link between *BRD4* and cataracts or related disorders. The following results provide evidence that *BRD4* is a causal gene for congenital cataracts. (1) BRD4 affected p62 and LC3B, key regulators of autophagy, which is a chief cellular mechanism associated with congenital cataracts. (2) BRD4 influenced CX43, a gap junction protein associated with oculodentodigital dysplasia, which is an extremely rare genetic condition that typically results in small eyes, underdeveloped teeth, and syndactyly and malformation of the fourth and fifth fingers. (3) BRD4 affected p62, which is not only a master regulator of ubiquitinated protein turnover via autophagy, but also causes Paget’s disease of bone [19]. Taken together, these results explain the patient’s syndromic symptoms.

Therefore, we inferred that *BRD4* gene defects are responsible for the common clinical features of our patients, including macrocephaly, short stature, and minor skeletal anomalies (shortened 3^rd^ and 4^th^ fingers and accessory navicular bones in both feet). In conclusion, *BRD4* may be a novel causative gene for an autosomal dominant syndromic congenital cataract disease.

## Supporting Information

S1 Table Summary statistics for whole exome sequencing (WES) in the studied family. (XLS)
