# Correction: Identification of a Novel Mutation in *BRD4* that Causes Autosomal Dominant Syndromic Congenital Cataracts Associated with Other Neuro-Skeletal Anomalies

**DOI:** 10.1371/journal.pone.0173757

**Published:** 2017-03-07

**Authors:** Hyun-Seok Jin, Jeonghyung Kim, Woori Kwak, Hyeonsoo Jeong, Gyu-Bin Lim, Cha Gon Lee

The second author’s name is spelled incorrectly. The correct name is: Jeonghyung Kim.

## References

[1] Jin H-S, Kim J, Kwak W, Jeong H, Lim G-B, Lee CG (2017) Identification of a Novel Mutation in *BRD4* that Causes Autosomal Dominant Syndromic Congenital Cataracts Associated with Other Neuro-Skeletal Anomalies. PLoS ONE 12(1): e0169226 doi: 10.1371/journal.pone.0169226 28076398 10.1371/journal.pone.0169226 PMC5226720

